# Evaluation of long-term care insurance pilot city policies in China: a cross-sectional study

**DOI:** 10.3389/fpubh.2025.1570794

**Published:** 2025-03-21

**Authors:** Kuan Du, Yuan Liu, Yiran Hu

**Affiliations:** Faculty of Military Health Service, Naval Medical University, Shanghai, China

**Keywords:** long-term care insurance, geriatric services, policy analysis, population aging, cross-sectional study

## Abstract

**Background:**

The acceleration of China’s aging process makes examining policy experiences from LTCI pilot cities highly significant for advancing the LTCI system in China and other emerging nations.

**Methods:**

Policy documents were gathered using Citespace 6.3.R1, with primary data sources obtained from official government websites. A comparative study of LTCI policies in pilot cities was conducted, focusing on policy support, participation scope, financing channels, benefit provision, care conditions, and management structures to evaluate the programs’ logic.

**Results:**

Pilot cities have developed distinct approaches in the LTCI development process. However, issues persist, including a singular financing channel, misalignment between benefit provision supply and demand, substandard care quality, and the need for improved management effectiveness.

**Discussion:**

Establishing a sustainable LTCI system requires implementing an independent financing mechanism and a standardized financial model, developing an efficient LTCI service supply framework, strengthening LTCI oversight and administration, and fostering the commercial insurance market.

## 1 Introduction

The rapid aging of population worldwide has increased the demand for geriatric care and medical treatment services, especially in the post–COVID-19 era (1). The issue of poor geriatric care services has received significant scholarly attention (2, 3). China has one of the world’s oldest populations, with over 296.97 million people aged 60 years or older by the end of 2023, representing 21.1% of the country’s total population. Among them, 216.76 million people are aged 65 years or older, accounting for 15.4% of the overall population, with a dependency ratio of 22.5% (4, 5). The prevalence of semi-disability and disability is rising, affecting approximately 35 million older adult people, or 11.6% of China’s older adult population, with a prevalence rate four times higher than that of the general population. The average duration of illness exceeds 8 years (6). Projections indicate that the number of older persons with disabilities in China may reach 46 million by 2035 and roughly 58 million by 2050 (7). The number of older persons requiring assistance for activities of daily living (ADLs) is rapidly increasing (8). However, conventional care given by senior family members is inadequate in China due to delayed childbearing and declining family sizes (9). Furthermore, the quality of such care is typically low, which has a negative impact on both caregivers’ well-being and older people’s rehabilitation (10).

Some high-income nations, like the Netherlands in 1968, the United States in the 1970s, Germany in 1995, and Japan in 2000, have implemented the LTCI program to address these issues (11, 12). The program provides monetary compensation and service protection for individuals requiring continuous care, such as those with cognitive impairments and disabilities, through institutionalized arrangements designed to mitigate the risks associated with population aging and establish a multilevel care protection system (13, 14). The World Health Organization (WHO) has recommended that China incorporate LTCI policies into its healthcare system to address older adult and disability care challenges (15–18). China’s LTCI initiative began in Qingdao in 2012, followed by phased LTCI pilots in 15 locations in 2016 and 2018 (19). By the end of 2022, trial cities had 331,000 care service professionals, 7,679 care service organizations, 16,992,000 LTCI members, and 12,080,000 individuals receiving treatment (20). However, the United Nations’ World Population Prospects report estimates that by 2050, over 500 million people in China aged 60 years or older, and by 2080, over 46% of the country’s population will be 65 years or older (21). The development of LTCI in China remains in its exploratory stage, and there is still a gap between the current available options and the need for older adult care (19). The Chinese government stated that it “will accelerate the construction of LTCI system” by 2025 in the Opinions on Deepening the Reform and Development of Older Adult Care Services, released in December 2024 (22).

In the future, the LTCI policy will be expanded by the Chinese government and applied across the country (22). However, China’s aging population, the swift increase in demand for aged care, and the global economy’s transition to slower growth present challenges in formulating an effective LTCI policy (10). Evaluating LTCI policies in pilot cities is essential for Chinese government officials and policymakers tasked with future decision-making (23). The main goal of this study is to enhance the analysis of China’s LTCI policy from both empirical and normative viewpoints. Limited research has been conducted on China’s LTCI policy, with most studies focusing on the financial mechanisms of the healthcare system and basic medical insurance for urban workers, which alleviates financial burdens on households and the government (24). This underscores the need for research evaluating the outcomes and effectiveness of China’s LTCI policy as a pilot initiative. Existing research on China’s LTCI policy primarily relies on conventional cognitive frameworks, focusing on conceptual constructions, operational mechanisms, and implementation strategies (25). This study examines the policy institutional framework of LTCI pilot cities in China to address these gaps. The second objective is to examine whether LTCI policies are facilitating the advancement of China’s healthcare insurance system. Current research indicates that LTCI has emerged as the “sixth insurance” within China’s social security framework and represents a significant policy initiative aimed at reforming the country’s healthcare system. The final objective of this study is to develop more progressive and socially pertinent LTCI policy recommendations for policymakers in China’s pilot cities.

### 1.1 Literature review

Since the launch of China’s LTCI policy pilot cities in 2016, an increasing body literature assessed this policy across three research phases: prior to 2016, the literature predominantly focused on the feasibility and necessity of LTCI, including analyses of LTCI systems in countries such as Germany and Japan, and outcomes of LTCI implementation in pilot cities like Qingdao (26–30). Between 2016 and 2018, the literature predominantly examined LTCI pilot regions in China and drew insights from international pilot experiences to optimize institutional design and LTCI implementation (31–34). Following 2018, as the development of LTCI accelerated, scholarly interest expanded to specific components of the policy, including examining the pilot policy and procedural aspects of system design, such as comparative analyses of the beneficiaries (35–37), financing mechanisms (38, 39), reimbursement standards (40), and service coverage (41). Additionally, studies have investigated the impacts on national finance and labor markets post-implementation of the LTCI policy, although comparative analyses of pilot regions remain limited (42). Current LTCI policy research is predominantly uniform. While interdisciplinary studies have examined LTCI from many viewpoints, it lacks a cohesive integration of theory and practice. Moreover, there is a paucity of studies evaluating the effectiveness of China’s LTCI pilot regions. Existing research on the efficacy of LTCI policies seldom employs the policy evaluation theory, often categorizing policy dimensions based on participating entities and the policies themselves, thereby lacking scientific theoretical frameworks and logical coherence.

### 1.2 Background

Chengdu, Qingdao, and Shanghai are among the initial pilot cities in China that have yielded favorable outcomes in the pilot phase, exemplifying the more progressive practices of China’s current LTCI system (43). Consequently, they also signify the future trajectory of LTCI development in China, which is dedicated to establishing a unified national LTCI framework. Thus, analyzing their experiences provides valuable insights into LTCI implementation nationwide. Consequently, drawing from the experiences of the pilot advanced regions, there exists a degree of universality in the analysis of the LTCI system’s implementation in China. Qingdao was the first region in China to implement the LTCI system and achieve comprehensive coverage among LTCI participants. In 2016, 15 cities, including Chengdu, Qingdao, and Shanghai, were designated as the initial pilot cities for the LTCI system in 2016. Shanghai has the highest number of individuals benefiting from LTCI in China, while Chengdu has the largest GDP and resident population in southwestern China. Furthermore, Chengdu had the highest total GDP and resident population in Southwest China and included both those with mild disabilities and those with dementia in its LTCI pilot program (44). Chengdu, Qingdao, and Shanghai represent pilot experiences in western, eastern, and northern China, respectively, and are equally indicative of the area. These cities also have a distinct advantage over other pilot cities regarding the accessibility of operational data pertaining on the LTCI system (45, 46).

Furthermore, Chengdu, Qingdao, and Shanghai are experiencing demographic aging, leading to a heightened need for older adult care; hence, the examination of LTCI holds substantial relevance for these three cities regarding geriatric care and healthcare provision. China’s seventh population census indicated that in 2020, individuals aged 65 and older in Chengdu, Shanghai, and Qingdao was 13.62, 16.3, and 14.2%, respectively, surpassing the national average of 13.5% (47). Consequently, examining the LTCI frameworks in these cities holds significant importance for other pilot cities in China.

## 2 Materials and methods

### 2.1 Data collection

The data for this study was sourced from the official websites of the Chinese government, including the National Health Insurance Bureau, the Social Security Bureau, and the municipal governments of Shanghai, Chengdu, and Qingdao. Two researchers conducted independent searches concurrently using keywords such as “long-term care insurance” and “long-term care.” The keywords included “long-term care insurance,” “long-term care,” and similar terms. The inclusion criteria required that the issuing entity be a governmental department from Shanghai, Chengdu, or Qingdao and that the content be significantly relevant to LTCI, primarily including laws, regulations, judgments, opinions, provisional measures, and notices. Exclusion criteria included policy interpretations, news articles, and other non-original policy materials, as well as keywords that lacked specific LTCI content within the policy texts. The publishing period was set from January 2016 to December 2024 to ensure the study’s uniqueness and continuity. Following the search, extraneous texts were manually removed and re-evaluated multiple times to ensure the reliability of the findings, guaranteeing that the collected texts accurately represented the development of LTCI policy.

### 2.2 Methods

The LTCI policy texts from Shanghai, Chengdu, and Qingdao were collected and analyzed using Citespace 6.3.R1, developed by Prof. Chaomei Chen. The objective was to conduct a comparative textual analysis to examine similarities and differences in the three cities’ LTCI systems, identify issues and their root causes, and provide detailed descriptions of LTCI policies concerning policy support, coverage scope, financing, payment, and care. This analysis highlights key practices, accomplishments, and weaknesses. Additionally, the study delineates LTCI policies in terms of policy support, participation breadth, financing, payment, and care while summarizing the principal practices, achievements, and deficiencies.

## 3 Results

### 3.1 Status of policy support

Since 2016, the number of LTCI-related local government documents released in Shanghai, Chengdu, and Qingdao, excluding informal government documents such as programs, stands at 32, 25, and 35, respectively. Chengdu has issued 12 local normative documents and four local working documents, Qingdao has released 15 local normative documents and 16 local working documents, while Shanghai has produced 21 local normative documents and six local working documents. Chengdu has comparatively fewer normative documents due to two primary factors: its pilot initiatives began later than those in Shanghai and Qingdao, resulting in inadequate preliminary research and predominantly non-governmental planning documents, and its aging demographic is relatively moderate, leading to unclear delineation of LTCI management responsibilities and a lower frequency of policy formulation (Table 1).

**Table 1:** Number of long-term care insurance documents issued in Shanghai, Chengdu, and Qingdao over the years.

City	2016	2017	2018	2019	2020	2021	2022	2023	2024	Amount
Shanghai	3	8	8	3	4	2	-	-	4	32
Chengdu	1	2	2	1	7	3	6	-	3	25
Qingdao	5	4	2	4	6	2	7	3	2	35

### 3.2 Participants

Chengdu, Qingdao, and Shanghai encompass both urban laborers and urban and rural inhabitants. The Chengdu LTCI participants include employees, residents, students, and children. Participants in provincial basic medical insurance are aligned to engage in Chengdu’s LTCI (48), and those enrolled in basic medical insurance under the single-build integrated model may opt to participate voluntarily (49). Chengdu and Qingdao impose no age restrictions on participants, while Shanghai mandates that resident participants be of at least 60 years (50, 51) (Table 2).

**Table 2:** Scope of long-term care insurance participants in Shanghai, Chengdu, and Qingdao.

City	Insurance coverage
Shanghai	Basic medical insurance for urban workers, basic medical insurance for urban residents over 60 years old
Chengdu	Basic medical insurance for urban workers, basic medical insurance for urban residents, Students and children groups
Qingdao	Basic medical insurance for urban workers, basic medical insurance for urban residents

### 3.3 Funding avenues

Shanghai relies solely on a single financing channel, exclusively dependent on the integrated medical insurance fund (52), whereas Chengdu and Qingdao utilize diversified financing avenues, including medical insurance funds, individual contributions, and government financial subsidies. In these cities, unit contributions are allocated directly from medical insurance funds, individual contributions are deducted from personal medical insurance accounts, and government financial subsidies come from medical insurance allocations.

Regarding financing standards, all three locations differentiate contributions based on employee and resident participation, except for Qingdao’s resident participation, which follows a fixed financing amount primarily based on the health insurance contribution base. A proportional financing method is applied, with the financing benchmark for employees exceeding that for residents (53). Shanghai exhibits the highest contribution base and ratio while exempting specific demographics, such as low-income groups, from contributions. Although Chengdu’s employee participation rate is lower than Qingdao’s, its resident participation is funded at a higher level. Chengdu’s employee financing structure also establishes varying individual contribution rates based on age and implements a differentiated subsidy policy, offering proportional subsidies for employees and fixed amounts for urban and rural residents, students, and children, including college students (49). The contribution base categorizes participants according to unique circumstances, employing both pass-through and hospitalization funding methods (Table 3).

**Table 3:** Financing of long-term care insurance in Shanghai, Chengdu, and Qingdao.

	Shanghai	Chengdu	Qingdao
Funding channels	Medical insurance pooled funds	Medical insurance fund, individual contributions, financial subsidies	Medical insurance fund, individual contributions, financial subsidies
Funding criteria	Employee participation: 0.5% of the employee health insurance contribution base and a quarterly transfer of funds from the employee health insurance pool fund; Resident participation: at a level slightly lower than the per capita financing level of employee participation, and quarterly transfers of funds from the resident medical insurance pool fund	Employee participation: a certain percentage of the employee health insurance contribution base (0.1% for those under 40 years of age, 0.2% for those over 40 years of age who have not retired, 0.3% for those who have already retired), 0.1%/year of the individual employee’s personal health care account contribution base, and 0.2%/month of the unit’s contribution base; Resident participation: individual contribution of 25 yuan/year, the government subsidizes a certain amount (≥ 60 years old 20 yuan/year, other 13 yuan/year)	Employee participation: 0.3%/month of the total employee health insurance contribution base, 0.2%/month of the individual contribution base of the employee’s personal health insurance account; government subsidy of 30 yuan/person/year; resident participation: no less than 10 yuan/person/year of the health insurance allocation, and no less than 20 yuan/person/year of the financial subsidy for health insurance

### 3.4 Provisions for benefits

All three cities have established their own disability assessment criteria. Chengdu and Qingdao follow the Trial Standards for Assessing Disability Levels published by the Health Insurance Bureau in 2021 (54), with Qingdao further differentiating criteria for individuals with disabilities and intellectual disabilities.

Chengdu and Qingdao assess four dimensions: daily living activities, mental status, perception and communication abilities, and social participation, which were subdivided into 19 and 22 indicators, respectively. The results were derived by aggregating the ratings of each dimension. Qingdao incorporates diagnostic assessments from medical institutions and specialists in its evaluation outcomes. Shanghai’s disability assessment criteria focus on self-care capability and illness severity, subdividing self-care capability into 50 specific indicators. The disability evaluation in Shanghai is predicated on the illness dimension, which markedly differs from that of Chengdu and Qingdao (55–57) (Table 4).

**Table 4:** Disability assessment status of long-term care insurance in Shanghai, Chengdu, and Qingdao.

	Shanghai	Chengdu	Qingdao
Assessment form	Shanghai Unified Needs Assessment Standards for Older Adult Care (Trial) Version 2.0	Norms for Comprehensive Assessment of Adult Disability in Chengdu Long-Term Care Insurance	Qingdao Care Needs Level Assessment Form for Disabled Persons, Qingdao Care Needs Level Assessment Form for Mentally Disabled Persons
Parameters for the assessment of incapacity	Self-care ability, severity of illness	Ability to perform activities of daily living, mental status, perceptual and social participation	Activities of Daily Living, Mental Status Assessment, Sensory Perception and Communication, Social Participation
Disability assessment level	Normal, care level 1 to care level 6	Intact, mildly disabled, moderately disabled (grades 1–3) and severely disabled (grades 1–3)	Disability 0–5; dementia is categorized as mild, moderate and severe
Number of beneficiaries (2020)	417,000	296,000	26,000

Regarding payment methods, Shanghai and Qingdao offer home care, institutional care, and daycare services, while Chengdu provides only home and institutional care and has yet to develop a community daycare model. Qingdao classifies care services into six types: home care, itinerant care, skilled nursing care, residential care, dementia care, and specialized care, with dementia care being the first of its kind in the country. In terms of financial support, the home care offerings in Chengdu and Shanghai closely resemble those of institutional care, although Qingdao’s institutional care services are comparatively more diverse.

In terms of financial support, Shanghai provides the highest payment sum among the three cities. Each city differentiates between employee and resident participants, implements a proportionate payment system, and establishes a payment cap. The home care model possesses the highest reimbursement rate. Concerning the payment limit, Chengdu and Qingdao explicitly delineate specific payment limits in their respective regulations, whereas Shanghai establishes the limit through negotiations between individuals and care providers, restricting the number of services and the duration of treatment. Payment norms and restrictions for employees in all three cities exceed those for residents, with the health insurance coordination fund significantly contributing to the disbursement of benefits (58) (Table 5).

**Table 5:** Status of long-term care insurance benefits in Shanghai, Chengdu, and Qingdao.

	Shanghai	Chengdu	Qingdao
Payment method	In-home care, community day care, institutional care	Home care, institutional care	Home care, institutional care, day care
Nursing service	Includes 27 items of basic life care and 21 items of clinical care	Home care: 18 items of basic care, 27 items of specialized care and assistive device services; Institutional care: basic care, specialized care and assistive device services are uniformly provided by the designated care institutions where they live	Home care: 10 items of living care and 32 items of medical care; Institutional care: 58 items, including basic living care, medical care services, cleaning and disinfecting of environmental goods, observation of diet and living conditions, and rehabilitation care.
Fund payments	Home care: 90%; day/institutional care: 85%	Employee participation: 75%; resident participation: 60%	Employee participation: 90%; first-tier contributors: 85%; second-tier contributors: 75%

### 3.5 Nursing assistance

All nursing care organizations have used the paradigm of agreement management for their regulation. Nursing institutions are categorized based on the services they offer, with Qingdao additionally classifying institutions based on the number of nursing beds, stipulating that only those with at least 30 nursing beds classify as residential nursing service institutions (59).

Concerning the evaluation of nursing care organizations, each city has established its own evaluation standards and defined unique criteria for assessment. The evaluation criteria in Qingdao encompass several elements, such as environment, management, services, and social impact, with care services assigned the highest importance, underscoring the divergent assessment priorities compared with those of Chengdu and Shanghai. Furthermore, in contrast to Chengdu and Qingdao, which employ standardized assessment criteria citywide, Shanghai’s evaluation metrics are determined by each district based on its own circumstances (60–62).

Concerning the nursing workforce, there exists a pervasive issue of inadequate staffing, predominantly comprising females, older adult individuals, and those possessing relatively poor educational qualifications. To resolve the disparity between the supply and demand for nursing staff and enhance the quality of nursing care, Chengdu has conducted multiple training programs for nursing personnel by engaging third-party institutions to elevate their professionalism. Conversely, Qingdao has released a policy paper regarding the training mechanism for nursing personnel, with the city’s medical insurance agency tasked with arranging and executing the training, thereby further standardizing the nursing personnel training process. Conversely, Shanghai has created its own regulations for the training of nursing personnel, according to local circumstances. For instance, Hongkou District has not only created a nursing talent pool but also elevated the pay of nursing personnel, resulting in Shanghai offering superior compensation and a more sufficient reserve of nursing staff (63–66) (Table 6).

**Table 6:** Long-term care insurance nursing facilities in Shanghai, Chengdu, and Qingdao.

	Shanghai	Chengdu	Qingdao
Type of organization	Medical institutions, nursing homes, community care facilities	Designated care institutions for residential care, designated care institutions for home-based care, designated training institutions, designated service institutions for assistive devices	Residential care service organizations, small-scale multi-purpose care service organizations, home-based care service organizations
Institutionally rated assessment programs	Districts set assessment standards	Comprehensive assessment program, special assessment program	Surrounding environment and transportation accessibility, basic management, care services, service performance, social influence and other additional items
Rating levels	One-star to five-star	Qualified, unqualified	Level A, B, C, D
Number of institutions (latest)	1,297	305	978

### 3.6 Management context

The Medical Protection Bureau serves as the responsible authority for LTCI management in all three regions, designating its subordinate unit as the operational and management organization. Chengdu’s management regulations only identify the competent authority and operational organization, without detailing the responsibilities of other government departments. In contrast, Qingdao and Shanghai provide explicit definitions of the responsibilities of caregiver training and fund management departments.

All three cities engage commercial insurance firms in LTCI payment processing and disability evaluations. In Shanghai and Chengdu, commercial insurers contribute to system development and management, while in Qingdao, they also oversee fund management (50, 51).

Chengdu employs a regional operation model, permitting each region to independently contract commercial insurers for the management and operation of their respective areas. In contrast, Shanghai and Qingdao employ an exclusive operation model, wherein a single insurer oversees the operation and management of the entire region. Shanghai has engaged commercial insurers to manage operational tasks for a limited duration, with the trial initiative only conducted in Putuo District (67).

Moreover, all three cities are actively investigating the development of LTCI platforms, intending to use the “Internet Plus” trend to enhance informatization and intelligent infrastructure. Shanghai has implemented online disability assessment processing via WeChat public accounts and mini-programs, alongside real-time monitoring of care service quality to enhance supervision. Qingdao and Chengdu have developed specialized mobile applications and other online tools to actively facilitate the online processing of disability assessments. Nevertheless, shortcomings persist in the development of information intelligence in the three cities, indicating substantial potential for enhancement (Table 7).

**Table 7:** Long-term care insurance administration in Shanghai, Chengdu, and Qingdao.

	Shanghai	Chengdu	Qingdao
Management unit	Medical security bureau	Medical security bureau	Medical security bureau
Fund management	Incorporated into the financial account of the Social Security Fund for unified management and earmarked funds	Incorporated into the financial account of the social security fund, with the implementation of two-line management, the establishment of detailed accounts for separate accounting, and earmarked for specific purposes	Two lines of income and two lines of expenditure, integrated into the management of financial accounts and earmarked for specific purposes
Operational model	Sub-district hosts	Exclusive contractor	Exclusive contractor

### 3.7 Challenges

#### 3.7.1 Increased strain on fund mobilization

Regarding the medical insurance fund, pertinent research indicate that the current sustainability of China’s urban workers’ medical insurance fund is robust, although the rate of increase in its sustainability has decelerated (68, 69). Certain studies have explicitly shown that the existing employee medical insurance framework may jeopardize the stability of the coordinated fund due to the LTCI model for employees derived from urban employees’ medical insurance (70). The yearly balance rate of the Chengdu fund was <10% in 2017, 2019, and 2021 (71–73). In Qingdao, the employee care fund amounted to 1.1 billion yuan in 2021, while the resident care fund totaled 145 million yuan (74). The overall expenditure of the care fund was approximately 677 million yuan, which seemed substantial at first glance; however, only 42,000 individuals actually received benefits (75). In 2021, the magnitude of Shanghai’s health insurance fund attained 522.9 billion yuan; however, if financed at the 1% ratio stipulated in Shanghai’s previous pilot scheme, the fund would only amount to 5.23 billion yuan, whereas the expenditure for establishing an LTCI system is significantly higher at 11.92 billion yuan (76). Certain researchers project that the expenditure for LTCI would amount to 224.134 billion yuan by 2030 and escalate to 618.120 billion yuan by 2050, indicating that the existing finance model is unable to accommodate the linear growth in the financial demands of LTCI (77). Consequently, the long-term viability of the basic medical insurance fund is under considerable strain.

#### 3.7.2 Disparity in benefit demand and supply

Chengdu and Qingdao exhibit disparities in reimbursement rates and maximum reimbursement limits for care services between employee and resident participants, with employee participants receiving more benefits than their resident counterparts. While this aligns with the contribution rates of the two participant categories, the cost of care for disabled individuals is determined by the severity of their condition rather than their employment or residency status. Simultaneously, the cost of nursing care services is relatively high, and those with lower incomes should benefit from an increased reimbursement threshold.

Chengdu and Shanghai have implemented the same evaluation methods and criteria for assessing individuals with disabilities and dementia. However, disability and dementia are distinct conditions. Disability primarily refers to physical impairments, requiring an emphasis on self-care and mobility, whereas dementia pertains to cognitive impairments, necessitating attention to thought processes and memory. Different evaluation criteria must be used to distinguish between the two. While Qingdao differentiates between them, its criteria for assessing dementia remain simplistic, relying solely on memory and orientation while neglecting aspects such as self-care and spatial awareness, which require further refinement.

#### 3.7.3 Inferior quality of nursing infrastructure

Chengdu, Qingdao, and Shanghai face a shortage of caretakers. In 2020, the proportion of impaired older adult individuals relative to the total older adult population in China was 16.6% (78). Based on this ratio, the estimated number of disabled older adult individuals in Chengdu, Qingdao, and Shanghai in 2020 was 353,600, 339,100, and 965,400, respectively. In the same year, the nursing staff in Chengdu, Qingdao, and Shanghai numbered approximately 20,000, 12,000, and 75,000, respectively, significantly below the stipulated 1:10 ratio of nursing staff to self-care individuals outlined in the “Norms for Job Setting and Staffing of Elderly Institutions” issued by the Department of Older Adult Services of the Ministry of Civil Affairs of China (79), revealing a substantial disparity with international standards (80).

Alongside the shortage of nursing personnel, there are inadequacies in the qualifications of nursing staff in Chengdu, Qingdao, and Shanghai. The quality of caregivers is inconsistent. The educational attainment of caregivers in these cities is predominantly low, with few holding a bachelor’s degree. There is also a deficiency in caregiving training and a lack of standardized training protocols. Furthermore, caregivers tend to be older, predominantly middle-aged women. The turnover rate among nursing personnel is high. Nursing work intensity is considerable, with many working over 10 h a day. However, nursing staff salaries are low, falling below the local average wage.

#### 3.7.4 Inadequate managerial efficacy

Collaboration with external organizations in the three regions is inadequate. In Shanghai, the medical insurance center primarily handles administrative management, with collaboration with commercial insurance firms in operational management occurring solely in Putuo District as part of a trial initiative. Additionally, in Qingdao and Shanghai, commercial insurance firms oversee the entire management process, from disability evaluation to benefit disbursement, without distinguishing between assessment and payment management. This may lead to conflicts of interest that could compromise the objectivity of evaluations.

Chengdu, Qingdao, and Shanghai also exhibit shortcomings in the informatization and intelligent development of LTCI (81). Despite the establishment of online information systems in all three cities, user feedback on their effectiveness has been unsatisfactory. In Chengdu and Qingdao, online LTCI services are limited to incapacity assessment apps, while in Shanghai, online functionalities are primarily used for monitoring care processes, resulting in a restricted expansion of online services. Additionally, many older adult individuals are unfamiliar with online operations, negatively affecting their experience with the program. The development of smart care equipment and institutions is progressing slowly and requires further improvement.

## 4 Discussion

### 4.1 Implementing viable funding strategies

The sustained advancement of LTCI primarily relies on the efficient allocation of expenditures. In China, the LTCI fund is still in its early stages, and a robust insurance funding system has yet to be established. In pilot cities, financing sources include medical insurance funds, contributions from individuals and organizations, financial subsidies, and allocations from social welfare and public welfare budgets. To reduce the financial burden of social insurance on businesses, some cities temporarily reallocated funds from the medical insurance fund during the pilot period (82–84). Certain cities provide financial subsidies based on economic development levels, making the health insurance fund the primary source of LTCI funding in China. We propose a comprehensive funding structure that incorporates government agencies, business entities, individuals, and third-party commercial insurers, drawing on models from other countries. This framework aims to create a balanced and systematic approach to the development of LTCI funds. Given China’s current and future economic trajectory, potential financing channels for LTCI can be categorized into five groups: government subsidies, health insurance funds, housing provident funds, social donations, and individual contributions. Social contributions encompass philanthropic donations, public welfare funds from lotteries, business financing, and other methods. The government must optimize fund allocation, improve the conversion rate of the health insurance fund, enhance fund autonomy, and ensure that funding levels correspond with economic development to meet the healthcare needs of the older adult population.

### 4.2 Perfecting LTCI service provision

The LTCI service system comprises nursing facilities, individual service providers, and management organizations. Currently, China faces a shortage of beds in healthcare and professional nursing facilities, leading to limited availability (85). Consequently, the care needs of disabled older adult individuals must be met by private nursing institutions. However, several obstacles hinder the development of private care facilities in China, resulting in outdated technology and facilities, as well as an uneven distribution of skilled workers. These challenges have limited the ability of private institutions to meet the needs of the older adult disabled population. The National Nursing Care Development Plan of China (2021–2025) recommends enhancing the integration of medical care and supporting the establishment of private nursing institutions (86). In the long term, encouraging private care institutions to participate in LTCI is a key objective and a crucial method to challenges of an aging population. The proficiency of caregivers is intrinsically linked to the efficacy of long-term care. Despite significant advancements in caregiver training in China, the country continues to confront challenges, including an aging workforce, inadequate educational attainment, elevated turnover rates, a lack of willingness to pursue the profession, and a rising demand for services at low wage levels (87). To resolve these issues, it is imperative to build professional nursing training institutions, develop a cohesive training system along with evaluation and assessment mechanisms, enhance faculty involvement and policy support, and elevate the professional quality and skill level of nursing personnel. Furthermore, an equitable system of occupational subsidies must to be established to cultivate a feeling of professional dignity, eradicate societal misconceptions and biases regarding the senior care profession, enhance the social standing of caregivers, and entice a greater number of young individuals into the field.

### 4.3 Enhancing the oversight and administration

The efficient functioning of LTCI necessitates a thorough and rigorous oversight and management structure. It is essential for the insured to establish explicit admission and discharge criteria, precisely evaluate health status and disability levels, and tackle the issues arising from inadequate or excessive service provision by employing a methodology that integrates random sampling with systematic monitoring and follow-up visits. Concerning service providers, the business license, staffing, and bed capacity should constitute the fundamental criteria for inclusion in LTCI. Local health insurance agencies must collaborate with experts from accredited healthcare organizations to perform a thorough evaluation of care providers’ qualifications and establish a systematic assessment framework. It is essential to enhance the oversight of the content and quality of institutional services. Moreover, pilot cities should employ varying criteria for evaluating LTCI quality, with quality management primarily falling under the jurisdiction of district-level health insurance agencies that report to municipal authorities. However, due to the diverse settings in which services are delivered, including private institutions, residences, and public hospitals, it is challenging for supervisory agencies to acquire accurate and actionable information. To resolve these issues, a robust quality assessment system must be implemented, utilizing known healthcare quality management methodologies. The system must encompass the evaluation of service receivers, credentials of service professionals, substance and timetable of services, a mechanism for gathering service-related data, and standardization of service procedures.

### 4.4 Developing robust commercial marketplaces

The Research Report on Long-Term Care Insurance by the Institute for Research on Aging at Fudan University indicates that China’s LTCI market exhibits notable deficiencies in medical and other specialized services, with demand from disabled older adult individuals significantly surpassing supply (88). China has initiated a pilot program that allows commercial insurance entities to engage in LTCI across 13 cities. Estimates indicate that the total demand for LTCI services for the older adult in urban areas of China in 2021 will approximate 1.4 trillion yuan, whereas protective measures, comprising household expenditures and policy-based LTCI and subsidies, will fall short of 500 billion yuan, resulting in a deficit of 921.7 billion yuan (82). In response to the increasing demand for older adult care services, it is essential to broaden the commercial older adult care insurance market and motivate more seniors to proactively select third-party older adult care insurance products, thereby alleviating the burden of policy-based insurance. Simultaneously, it is crucial to proactively investigate the development of a multilayer pension service system that amalgamates home, community, and institutional assistance, while persistently enhancing China’s LTCI market.
